# Hospital and Physician Group Practice Participation in Prior and Next-Generation Value-Based Payment Programs

**DOI:** 10.1001/jamanetworkopen.2024.0392

**Published:** 2024-02-26

**Authors:** So-Yeon Kang, Gerard Anderson

**Affiliations:** 1 Department of Health Management and Policy, Georgetown University School of Health, Washington, DC; 2 Department of Health Policy and Management, Johns Hopkins Bloomberg School of Public Health, Baltimore, Maryland

## Abstract

This cohort study examines whether prior direct or indirect participation in the Centers for Medicare & Medicaid Innovation Bundled Payments for Care Improvement (BCPI) Initiative was associated with their participation in the next generation of the program.

## Introduction

Getting hospitals to participate in value-based payment reform voluntarily is a challenge.^1^ One of the largest voluntary payment models run by the Center for Medicare & Medicaid Innovation (CMMI) is the Bundled Payments for Care Improvement (BPCI) Initiative.^2^ Prior studies have focused primarily on early adopter hospitals and did not consider the role of dropout history and indirect exposure through physicians.^3,4,5,6^ This study examines whether prior participation in the BPCI Initiative, direct or indirect through physician group practices (PGPs), was associated with their participation in the next generation of the program (BPCI Advanced).

## Methods

In this retrospective cohort study, acute care hospitals eligible for BPCI Advanced were included. The CMMI’s BPCI and BPCI-Advanced analytic files from October 1, 2013, to December 31, 2021, and a 20% random sample of Medicare inpatient claims data in 2017 were used to identify hospitals’ BPCI history and BPCI-Advanced participation. The Medicare Cost Reports and American Hospital Association Annual Survey supplemented the data. The study was exempt from institutional review board at the Johns Hopkins Bloomberg School of Public Health because human participants were not involved. This study followed the STROBE reporting guideline for observational studies.

The outcomes of interest included hospitals’ participation (yes or no) and participation type (direct or indirect through PGPs) in BPCI Advanced. The key explanatory variable, hospitals’ prior BPCI engagement, had 5 mutually exclusive categories: (1) early BPCI adopters, (2) indirect participation through PGPs after dropout, (3) indirect participation only, (4) dropout only, and (5) no prior BPCI exposure.

Covariates encompassed hospitals and communities’ characteristics. Adjusted multilevel mixed-effects logistic regression was performed to account for interactions between hospital- and community-level variables. Regression was repeated for hospital subgroups based on teaching and ownership status (eMethods in Supplement 1). Analyses were performed using Stata, version 17 (StataCorp). All *P* values were from 2-sided tests, and results were deemed statistically significant at *P* < .05.

## Results

**Figure 1.**
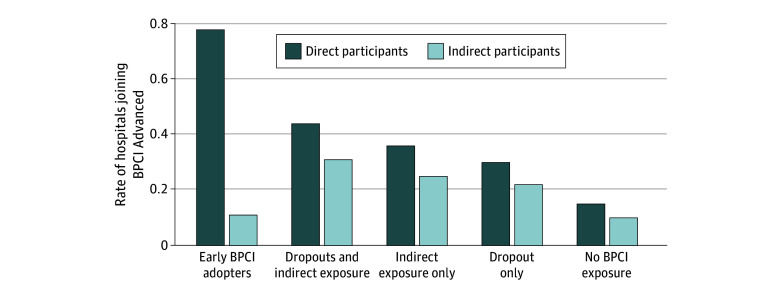
Hospital Participation Rates in Bundled Payments for Care Improvement (BPCI) Advanced by BPCI Experience Type Early BPCI adopters: hospitals that applied to participate in the BPCI Initiative, transitioned to the risk-bearing phase, and completed the program. Dropout: hospitals that applied to participate in the BPCI Initiative but withdrew their participation before or during the risk-bearing phase. Indirect participation through physician group practices (PGPs): hospitals that did not directly participate in the BPCI Initiative but were affiliated with participating PGPs. These hospitals were identified through the combination of BPCI Initiative flags in Medicare inpatient claims data and BPCI Initiative analytic files.

**Figure 2.**
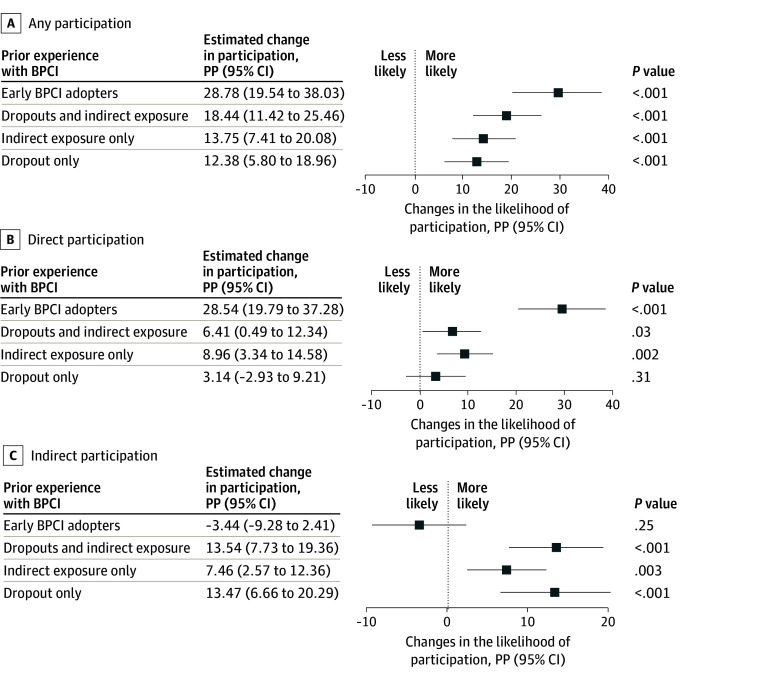
Adjusted Associations of Hospitals’ Prior Bundled Payments for Care Improvement (BPCI) Experience With Their Participation in BPCI Advanced by Participation Type Estimates are derived from a multilevel logistic regression model accounting for clustering by community based on core-based statistical area. Covariates included (1) size: the size of the hospital based on the number of beds, the volume of BPCI Initiative–eligible episodes, the proportion of inpatient cases eligible for the 10 most common BPCI Initiative episodes out of the total inpatient volume for each hospital; (2) structure: for-profit hospitals measuring potential resource availability, government hospitals, members of a health system; (3) innovativeness: teaching hospitals based on the resident to bed ratio, total research cost of the hospital; and (4) enabling resources: total profit margin, use of skilled nursing facilities, home health agencies, and remote patient monitoring. In addition, the Medicare case-mix index of the hospital was included to control for differences in the severity and resource intensity of admitted patients. Community-level covariates were derived from the hospital-level characteristics (eg, the prevalence of teaching hospitals). PP indicates percentage points.

Our sample consisted of 3248 hospitals eligible for BPCI Advanced. Of these, 37.8% were teaching hospitals, 24.9% were for-profit hospitals, and 77.6% were affiliated with a health system. The median number of beds was 132 (IQR, 57-260). The sample had early BPCI adopters (7.7%), indirect participants after dropout (17.5%), solely indirect participants (34.6%), and dropout history alone (6.3%) as well as hospitals without BPCI exposure (33.9 %), which were generally small, nonteaching, and independent institutions. Hospitals with prior BPCI experience were more likely to join the BPCI-Advanced initiative (Figure 1). After controlling for covariates, early BPCI adopter hospitals had a higher likelihood of participation than those without prior experience (28.78 [95% CI, 19.54-38.03] percentage points), followed by indirect participants after dropout (18.44 [95% CI, 11.42-25.46] percentage points), those with solely indirect participation (13.75 [95% CI, 7.41-20.08] percentage points), and those with dropout history alone (12.38 [95% CI, 5.80-18.96] percentage points) (Figure 2). The regression results held within the subgroups as specified.

Hospitals with a dropout history were more likely to choose indirect vs direct participation through PGPs (3.14 vs 13.47 percentage points), while indirect exposure–only hospitals had a similar probability of joining either directly or indirectly (8.96 vs 7.46 percentage points) (Figure 2). Early BPCI adopter hospitals were more likely to rejoin as direct than indirect participants (28.54 vs −3.44 percentage points).

## Discussion

Two-thirds of hospitals eligible for BPCI Advanced had experience with BPCI and were more likely to join subsequent programs. Hospitals’ indirect program involvement via PGPs and dropout history were associated with the increased likelihood of participation in the subsequent program. The findings reveal a persistent challenge in engaging hospitals without prior exposure to new value-based payment initiatives relying on voluntary participation. The CMMI may consider promoting participation among affiliated physician groups if the hospitals choose not to directly participate. Limitations include that our study did not examine episode-level participation (eg, clinical vs surgical) or establish a causal effect of past on future participation behavior.

## Notes

Supplement 1.
eMethods.

Supplement 2.
Data Sharing Statement
