# Supplementary material for: Hospital and Physician Group Practice Participation in Prior and Next-Generation Value-Based Payment Programs
Source: JAMA Netw Open. 2024 Feb 26;7(2):e240392. doi: 10.1001/jamanetworkopen.2024.0392 (PMC10897743; doi:10.1001/jamanetworkopen.2024.0392)
Supplement: Supplement 1. — eMethods. [file jamanetwopen-e240392-s001.pdf]

Supplementary Online Content

Kang SY, Anderson G. Hospital and physician group practice participation in prior and next-generation value-based payment programs. *JAMA Netw Open.* 2024;7(2):e240392. doi:10.1001/jamanetworkopen.2024.0392

eMethods.

This supplementary material has been provided by the authors to give readers additional information about their work.

© 2024 Kang SY et al. *JAMA Network Open.*

eMethods.

Study Sample

We focused on general short-term acute care hospitals as defined in the 2018 Medicare cost report data and excluded new hospitals that opened after 2018. Hospitals with other unique payment methodologies were excluded from the BPCI program and this analysis. These included: Prospective Payment System (PPS)-Exempt Cancer Hospitals, Inpatient Psychiatric facilities, Critical Access Hospitals, hospitals in Maryland, and hospitals participating in the Rural Community Hospital Demonstration and Participant Hospitals in the Pennsylvania Rural Health model.

Variables

Outcome variables

The outcome of interest is hospitals' participation at any degree and type of participation (direct and indirect participation through PGPs) in BPCI-Advanced in Period 2. In BPCI-Advanced, formally participating hospitals bear financial risks for BPCI-Advanced episodes through a direct agreement with CMS or with third-party conveners (i.e., other participating hospitals or consulting firms) that have direct agreements with CMS on behalf of the hospitals. Lists of hospitals directly participating in BPCI-Advanced from 2018 to 2021 were obtained from the CMS analytic files. These hospitals were coded as direct participants in BPCI-Advanced. Other hospitals can collaborate with participating PGPs by allowing their physicians to initiate BPCI episodes within their hospitals. These hospitals were coded as indirect participants in BPCI-Advanced. Both direct and indirect participation was coded as any participation to capture their engagement in the BPCI ecosystem. Hospitals without any record of direct or indirect participation in the public files were coded as no participants.

Explanatory variables

The key explanatory variable is the hospital's experience with the BPCI initiative. Prior experiences in the payment reform were defined as the combination of two factors: type of engagement in BPCI (direct participation vs. indirect participation via PGPs), and the dropout history as an indicator of their interest in the payment model. Hospitals that did not apply to participate in BPCI but were affiliated with participating physician group practices, as identified through the combination of BPCI flags in Medicare inpatient claims data and BPCI analytic files, were classified as having indirect participation.

Direct participation was identified using CMS BPCI analytic files from 2013 to 2018. Hospitals publicly reported in at least one model period are defined as participants of the BPCI model. Among these, hospitals not reported in subsequent years were defined as dropouts.

Indirect participation via PGPs was identified using a 20% random sample of Medicare fee-for-service inpatient claims data in 2017. BPCI episodes were attributed to a participating hospital when the hospital was the provider organization listed on the inpatient claim.[12] Conversely, BPCI episodes were attributed to a participating PGP when the attending or operating physician of the inpatient claim was a member of the group, even though the provider hospital was not participating in BPCI.[12] These non-participating hospitals' indirect exposure through physicians to BPCI was identified with the bundled payment claims code.

Other covariates

Other covariates encompass predisposing characteristics of both hospitals and communities. In the traditional diffusion theory, adopter characteristics that influence the adoption decision can be categorized into three groups: organization size, organization structure, and organizational innovativeness.[13-16] Based on these theoretical frameworks, a set of covariates was identified as the following: (1) size: the size of the hospital based on the number of beds, the volume of BPCI eligible episodes. Also, to comprehend hospitals' potential dependence on services eligible for

© 2024 Kang SY et al. *JAMA Network Open.*

BPCI episodes, we calculated the proportion of inpatient cases eligible for the ten most common BPCI episodes out of the total inpatient volume for each hospital; (2) structure: for-profit hospitals measuring potential resource availability, government hospitals, members of a health system (vs. independent hospital) ; (3) innovativeness: teaching hospitals (i.e., hospitals with major and minor teaching engagement based on the resident-to-bed ratio), total research cost of the hospital ; (4) enabling resources: total profit margin, use of skilled nursing facilities, home health agencies, and remote patient monitoring. In addition, the Medicare case-mix index of the hospital was included to control for differences in the severity and resource intensity of admitted patients.

Statistical analysis

Adjusted multilevel logistic regression was performed because explanatory variables include different levels (hospital level and CBSA level), and there can be interactions of variables between levels. Along with the set of community-level fixed effect variables, hospital-level variables were included. The regression results were converted to average marginal effects to estimate the variable’s probability of participation in BPCI-Advanced upon a unit change in explanatory variables. The variance inflation factor was calculated for each explanatory variable to examine multicollinearity among variables. Two-tailed statistical tests were used and considered significant at $\alpha=.05$. Standard errors were clustered at the community level. All analyses were performed using software packages Stata version 17 (StataCorp LLC).

© 2024 Kang SY et al. *JAMA Network Open.*
