# Supplementary material for: Hospital and Physician Group Practice Participation in Prior and Next-Generation Value-Based Payment Programs
Source: JAMA Netw Open. 2024 Feb 26;7(2):e240392. doi: 10.1001/jamanetworkopen.2024.0392 (PMC10897743; doi:10.1001/jamanetworkopen.2024.0392)
Supplement: Supplement 2. — Data Sharing Statement [file jamanetwopen-e240392-s002.pdf]

Data Sharing Statement

Kang. Hospital and Physician Group Practice Participation in Prior and Next-Generation Value-Based Payment Programs. *JAMA Netw Open*. Published February 26, 2024. doi:10.1001/jamanetworkopen.2024.0392

Data
Data available: No

Additional Information
Explanation for why data not available: Our study includes Medicare Claims data, which comes with a restrictive data user agreement prohibiting data sharing.
